# Impacts of Urbanization and Habitat Characteristics on the Human Risk of West Nile Disease in the United States

**DOI:** 10.3390/biology14030224

**Published:** 2025-02-20

**Authors:** Jian Ma, Nuo Xu, Ying Xu, Zheng Y. X. Huang, Chuanwu Chen, Yingying X. G. Wang

**Affiliations:** 1 School of Life Sciences, Nanjing Normal University, Nanjing 210023, China; jianma@tsinghua.edu.cn (J.M.); 221202148@njnu.edu.cn (N.X.); 231202153@nnu.edu.cn (Y.X.); zyxhuang@njfu.edu.cn (Z.Y.X.H.); chencw@nnu.edu.cn (C.C.); 2 Vanke School of Public Health, Tsinghua University, Beijing 100084, China; 3 School of Life Sciences, Nanjing Forestry University, Nanjing 210023, China; 4 Department of Biological and Environmental Science, University of Jyväskylä, FI-40014 Jyvaskyla, Finland; 5 One Health Institute, School of Veterinary Medicine, University of California, Davis, CA 95616, USA

**Keywords:** West Nile virus, landscape factor, *Culex pipiens*, *Culex tarsalis*, climatic factors, host community composition

## Abstract

Since its initial identification in 1999, West Nile virus has spread rapidly throughout North America, exhibiting high spatial heterogeneity. Previous studies exploring the spatial patterns of the human risk of West Nile Disease (WND) in the United States have demonstrated the important roles of landscape and climatic factors. However, relatively few studies have endeavored to elucidate the effects of habitat fragmentation on WND risk, though it has been considered to affect disease risk through its influence on host community composition, vector abundance and human–vector–host interactions. In this study, we investigated and compared the effects of landscape factors, with a particular focus on habitat fragmentation, on the human risk of WND in the eastern and western United States. Our results demonstrated that landscape factors exhibited significant relationships with disease risk in both regions, while their effects could vary between the regions. Generally, urbanization was positively correlated with the WND risk in both regions, while the fragmentation indices of developed areas showed negative correlations only in the east. In contrast, forest area positively correlated with WND risk in the west, while a negative relationship was found in the east. The fragmentation indices of natural areas in both regions were generally positively associated with WND risk. These differences may be due to the differences in vector species and related processes (host-related or vector-related) between the two regions. With ongoing environmental change, this study provides new insights into understanding the risk factors for WND in the United States and the effects of habitat fragmentation on animal disease risk.

## Simple Summary

As one of the most important zoonotic pathogens, West Nile virus (WNV) has spread throughout the United States, posing a severe threat to both human and animal health. Due to the lack of human vaccines, understanding the epidemiology and risk factors for West Nile Disease (WND) is crucial for disease prevention and control. Previous studies have identified many landscape factors as the risk factors for WND. However, relatively few studies focused on habitat fragmentation, which has been proven to affect the risk of several vector-borne diseases. Therefore, in this study, we explored the associations of landscape factors, with a particular focus on habitat fragmentation, with human WND risk in the United States. We also compared the risk factors between the eastern and western regions. We found that the fragmentation of natural areas (such as forests and wetlands) was generally positively associated with WND risk in both regions, while the fragmentation of developed areas showed negative correlations only in the eastern region. In the context of ongoing land use change, this study not only provides new insights into the risk factors for WND but also sheds light on the effects of habitat fragmentation on animal disease risk.

## 1. Introduction

West Nile virus (WNV), belonging to the family Flaviviridae, genus *Flavivirus*, is one of the most significant zoonotic pathogens in the United States. Since its initial identification in New York in 1999, the virus has spread throughout the continental United States, posing a severe threat to both human and animal health [1,2]. As of 2023, the Center for Disease Control and Prevention (CDC) in the United States has reported a total of 59,141 human cases of West Nile Disease (WND), including 30,442 cases (48.8%) of neuroinvasive disease and 2820 deaths (4.6%) (www.cdc.gov/west-nile-virus/data-maps/historic-data.html, accessed on 25 May 2023). In addition, WNV has resulted in at least 28,000 equine cases and the deaths of over 300 bird species in the Americas, leading to significant population declines in at least 23 bird species [2,3]. Studies have indicated an annual economic loss of up to 56 million US dollars in the United States due to the WNV [2]. Beyond the United States, the largest WNV outbreak recorded in Europe occurred in 2018, and the WNV has posed an ongoing threat in many areas around the world [4]. Considering the current lack of effective treatment methods and human vaccines available for WNV [2,5], studying the risk factors associated with its transmission is of crucial importance for the future monitoring and control of WNV.

WNV usually propagates in sylvatic cycles, where birds are considered the principal host species, while humans and other vertebrates are usually incidental hosts [6]. Although mosquitoes within the genus *Culex* are the primary vectors for WNV in North America [7], the main *Culex* species and their contributions to WNV circulation vary between different regions [8,9]. A previous review concluded that *Cx. pipiens* plays the most important roles in Northeastern, mid-Atlantic and central United States, while *Cx. quinquefasciatus* is the most important vector species in the southeastern region [8]. In the western United States, *Cx. tarsalis* serves as one of the most important vector species due to its high abundance and competence for WNV. In addition to *Cx. tarsalis*, *Cx. pipiens* and *Cx. quinquefasciatus*, respectively, in the northern part and southern part of the western region, also contribute to WNV transmission [10]. It has been suggested that hundreds of bird species can be infected by WNV; however, the competence for the virus showed a great variation among these species. Some species of Passeriformes, Charadriiformes and Strigiformes can have a higher competence, while the Columbiform, Pelecaniform, Psittaciform and Galliform orders are considered less competent for virus transmission [11].

Many studies have been conducted to explore the spatial patterns of WND risk in the United States, and these studies have identified a variety of landscape factors that can affect WNV transmission. For example, a previous study examined the spatial pattern of human WND incidence and found that disease risk was positively correlated with urban land cover in the northeastern United States, while the WND risk was positively correlated with agricultural land cover in western regions [12]. Moreover, previous research also demonstrated that the human WND incidence was higher in areas with greater coverage of irrigated cropland in north-central Colorado [13] and in rural areas close to wetlands and ponding areas in South Dakota [14]. Additional studies have suggested that the variations in landscape factors between the regions might be attributed to the differences in mosquito species that contribute to WNV transmission [9].

In addition to landscape factors, climatic factors can also affect WNV transmission by influencing the survival of the virus and vectors, as well as the hosts’ development, reproduction and behavior [15,16,17]. For example, in North Dakota and Minnesota, high temperature was found to be associated with a higher prevalence of WNV in mosquitos and more human cases [18], as higher temperatures can facilitate virus replication in mosquitoes, prolong mosquito breeding season, and increase the abundance of mosquitoes [19,20]. Moreover, elevated temperatures can also increase the bite rates and frequency of mosquitoes, thereby increasing the WNV transmission risk [21,22]. Compared to temperature, the effect of precipitation on WNV transmission is not consistent between different regions [23]. For instance, by dividing the United States into eastern and western regions, Hahn et al. investigated and compared the influence of climate factors on human WND incidence in the eastern and western United States and found that the impact of precipitation on disease risk differed between the two regions [9].

These risk factor analyses have greatly deepened the understanding of the transmission mechanisms of the WNV, but gaps remain. For example, many studies have demonstrated that habitat fragmentation, as an important landscape factor, can be closely related to the risk of infectious animal diseases due to its effects on host movements, community composition, vector abundance, and behavior [24,25]. However, few studies have explored the role of habitat fragmentation in WNV transmission [26,27]. In this study, the potential landscape and climate factors related to the human risk of WND were investigated at the county level, with a focus on the impact of habitat fragmentation. Following a previous study [9], this study divided the United States into eastern and western regions and compared the effects of risk factors between the two regions.

## 2. Materials and Methods

### 2.1. Study Area and Disease Data

**Figure 1:**
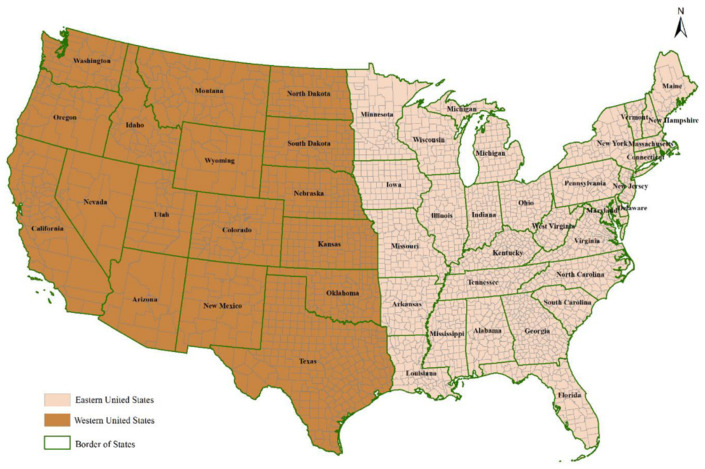
Map of West Nile virus research areas in the United States.

The study area included the contiguous United States. Following a previous study [9], the study area was divided into eastern and western regions along the longitude of 95–100°, including 32 states in the eastern region and 17 states in the western region (Figure 1, Table S1). County-level data on human WND cases (including both neuroinvasive and non-neuroinvasive cases) from 2003 to 2021 were collected from the CDC without any accompanying personal identifying information.

### 2.2. Data of Predictors

This study considered a variety of landscape and climatic predictor variables (Table 1). Regarding landscape factors, the land-use cover maps were collected from the National Land Cover Database (NLCD) [28]. The present study focused on 10 land cover classes, 5 of which were related to human uses: developed open space (class 21), low-intensity developed space (class 22), medium-intensity developed space (class 23), high-intensity developed space (class 24), and cultivated crops (class 82). The other five classes were related to natural areas, including deciduous forest (class 41), evergreen forest (class 42), mixed forest (class 43), grassland (class 71), and woody wetlands (class 90). For each land cover class, the total area within the county (CA), the proportion of land cover area relative to the total county area (PLAND) and the number of patches (NP) were calculated in Fragstats 4.2. CA and NP were used to calculate the fragmentation index (FN), which was defined as the number of patches divided by the total area, representing the degree of fragmentation of a specific class within the landscape [29]. For each year, landscape factors were calculated based on the land-use cover maps for the nearest year.

For climatic factors, this study considered the annual mean temperature (TemMean), annual mean precipitation (PreMean), mean temperature of the driest quarter (BIO9), mean temperature of the warmest quarter (BIO10), mean temperature of the coldest quarter (BIO11), precipitation of the driest quarter (BIO17), precipitation of the warmest quarter (BIO18), and precipitation of the coldest quarter (BIO19). These climatic variable maps were collected from the WorldClim (https://worldclim.org, accessed on 15 January 2023) version 2, where these variables are the average for the years 1970–2000. We calculated, for each county, the mean value of these variables. The preprocessing and calculation of all predictors were conducted in ArcGIS 10.5 and Fragstats 4.2.

### 2.3. Statistical Analysis

Generalized linear mixed models (GLMMs) were employed to test the relationships between the predictors and human WND cases. We applied a negative binomial distribution to reduce the overdispersion of residuals. The state and year were included as random factors to control for potential differences between states and years. Prior to implementing the GLMM, all predictor variables were scaled to have a mean of 0 and a standard deviation of 1.

We first conducted single-variable analyses to examine the correlations between WND cases and each predictor, and predictors with a *p*-value < 0.05 were identified as potential risk factors. We then evaluated the multi-collinearity of these potential risk factors by calculating the pairwise correlation coefficients (*r*). We then constructed multi-variable regression models following a full-as-possible model-building strategy, where only one of those highly correlated variables (*r* > 0.65) was retained in any candidate subset model to make sure that all variables in any model had a variance inflation factor (VIF) smaller than 5. Model averaging was then performed by ranking all candidate models based on the corrected Akaike information criterion (AICc) and derived Akaike weights (*w_i_*). Models with an ΔAICc < 2 were considered competing models and were averaged [30]. In both single- and multi-variable analyses, the area of the county (AREA, log-transformed) and the human population size (population, log-transformed) were retained in the model to control for the effects of area size and human population. Models were constructed in R 4.2.2 using the package *lme4* (version 1.1.36) [31] and *MuMIn* (version 1.47.5) [32], and model validation was performed using package *DHARMa* (version 0.4.6) [33].

## 3. Results

### 3.1. Single-Variable Regression Analyses

The single-variable regression analyses identified a variety of potential risk factors for the WND risk in both the eastern and western United States (Table 2). For landscape factors, the human WND risk was generally positively correlated with the proportion of developed area (PLAND21, 22, 23, and 24) within the county, while negatively correlated with the fragmentation indices of open and medium developed areas (FN21 and FN23, respectively). The human WND risk was generally negatively correlated with the proportion of forests (PLAND 41, 42 and 43), except for the proportion of evergreen forest (PLAND42), which showed a positive effect in the eastern United States. In contrast, all the fragmentation indices of forests were positively correlated with human WND risk. In addition, the proportions of grasslands and croplands (PLAND71 and 82) were positively correlated with WND risk in both the western and eastern United States. The proportion of woody wetlands (PLAND90) showed a positive correlation with WND risk in the eastern region and a negative correlation in the western region. In both the two regions, the WND risk was negatively associated with the fragmentation index of grasslands (FN71).

For climate factors, the single-variable regression analyses demonstrated that in both the eastern and western United States, the WND risk was positively correlated with the mean temperature of the warmest quarter (BIO10), while it was negatively correlated with the annual mean precipitation (PreMean) and the mean precipitation of the warmest quarter (BIO18). The mean precipitation of the coldest quarter (BIO19) and the mean temperatures of the driest quarter (BIO9) and the coldest quarter (BIO11) were negatively correlated with the WND risk in the western United States, while these factors were positively associated with the WND risk in the eastern region. In addition, WND risk in the western region was also positively correlated with the annual mean temperature (TemMean), and negatively correlated with the precipitation of the driest quarter (BIO17).

### 3.2. Multi-Variable Regression Analysis

In both the eastern and western United States, only the top model was competitive (*w_i_* = 0.96 and *w_i_* = 0.99, respectively, for models of the eastern and western regions). In both regions, we found little evidence of overdispersion for the residuals of the top model (Figure S1, *p* = 0.648 and 0.096, respectively, for the eastern and western regions). The conditional adjusted R^2^ and marginal adjusted R^2^ for the top model in the eastern United States were, respectively, 0.653 and 0.386, while 0.759 and 0.420 for the western region.

The results of multi-variable regression analyses (Table 3, Figure S2) showed that in both the eastern and western regions, the WND risk was positively correlated with the mean temperature of the warmest quarter (BIO10) and negatively correlated with the mean precipitation of the warmest quarter (BIO18). In addition, the WND risk in the western region was positively correlated with the annual mean temperature (TemMean), while it was negatively correlated with the annual mean precipitation (PreMean).

In addition to climatic factors, the human WND risk in the eastern United States was also significantly positively correlated with the percentage of area covered by medium-intensity developed land in urban areas (PLAND23) and grassland (PLAND71), while negatively correlated with the percentage of area covered by deciduous forest (PLAND41) and evergreen forest (PLAND42). Additionally, the WND risk in the eastern United States was negatively correlated with the fragmentation indices of low- and medium-intensity developed land (FN21 and FN23) and grassland (FN71), while positively correlated with the fragmentation indices of deciduous forest (FN41) and mixed forest (FN43).

In the western United States, the results of multi-variable regression models (Table 3, Figure S3) showed that the human WND risk was positively correlated with the percentage of area covered by low- and high-intensity developed land (PLAND22 and PLAND24, respectively), deciduous forest (PLAND41), as well as cropland (PLAND82) and woody wetland (PLAND90), while negatively correlated with the percentage area covered by mixed forest (PLAND43). In addition, the fragmentation indices of mixed forest (FN43), cropland (FN82) and woody wetland (FN90) were also positively associated with WND risk.

## 4. Discussion

The present study investigated the potential landscape and climate factors associated with the human risk of WND in the contiguous United States, with a particular focus on the impacts of habitat fragmentation. The findings demonstrate that both climatic and landscape factors can affect the human WND risk in the United States, but the risk factors and their effects may differ between the eastern and western regions.

Our multi-variable regression analyses showed that human WND in the eastern United States was positively correlated with the percentage area of developed area (PLAND 23), while negatively correlated with the percentage areas of forests (PLAND 41, 42). It has been suggested that *Cx. pipiens* and *Cx. quinquefasciatus* are the most important vector species for WNV in the eastern region [10]. Both of the two species often breed in contaminated or nutrient-rich artificial containers, such as rain gutters, urban drainage pipes, and ditches. Abandoned buildings or swimming pools can also facilitate the growth of *Cx. pipiens* populations [34,35]. Therefore, urbanization promotes the WND risk in the eastern region. These results were consistent with a previous study, which also found similar correlations in the eastern United States by analyzing WND data from 2002 to 2008 [12]. However, in contrast to this study that found negative relationships between WND risk and developed areas in several western regions, our analyses found the percentage of the developed area (PLAND 22 and 24) was positively correlated with WND risk in the western United States. Moreover, we also found that the percentage areas of deciduous forest, cropland and woody wetland were positively correlated with the WND risk in the western United States. These different associations between the eastern and western United States might be caused by the difference in vector species between the two regions. In addition to *Cx. pipiens* and *Cx. quinquefasciatus*, *Cx. tarsalis* can also contribute to the transmission of the WNV in the western region [10]. However, unlike *Cx. pipiens* and *Cx. quinquefasciatus*, *Cx. tarsalis* tends to thrive in natural water pools or irrigated croplands [10], which may explain the positive effects of forests, croplands and wetlands on the WND risk in the western region.

The fragmentation indices sometimes showed different relationships with the WND risk, compared to the effects of the proportion of land cover areas. Here, we found that the fragmentation indices of developed areas were only significantly correlated with the WND risk in the eastern region. Fragmentation of developed areas, particularly low- and medium-intensity developed areas, may limit their capacity to promote the abundance of *C. pipiens*, the main vector in the eastern region. In contrast, none of any fragmentation indexes of developed areas exhibited a significant relationship with the WND risk in the western region. We considered that *C. pipiens* may be less important in WNV transmission in the west than in the east, as another mosquito species, *C. tarsalis*, also contributes to virus transmission. Simultaneously, greater human–vector contact in fragmented developed areas also counteracts the negative effect of fragmentation on mosquito abundance. In both the eastern and western regions, the fragmentation indices of natural areas (i.e., deciduous and mixed forests in both regions and woody wetlands in the western region) exhibited positive correlations with the WND risk, which might be attributed to their effects on the bird host community. It has been suggested that less competent hosts for the WNV are usually more vulnerable to species loss and have a higher local extinction risk when the community disassembles [36,37]. Greater forest fragmentation can cause the decline in community biodiversity, which increases the likelihood that these less competent hosts will go extinct, subsequently promoting the abundance of competent hosts due to predation or competition release [8]. Moreover, human activity may be increased in fragmented forests [38,39], facilitating contact between humans and infected mosquitoes and thereby elevating the WND risk.

In terms of climate factors, the results indicate that temperature, particularly the mean temperature during the warmest quarter, strongly promotes the risk of WND in the United States. Conversely, greater precipitation during the warmest quarter may be associated with a decrease in the risk of WND. Increases in temperature and decreases in precipitation can influence the behavioral activities of hosts and vectors involved in WNV transmission, as well as their environmental conditions. Higher temperatures favor virus replication, promote mosquito breeding and development, increase the frequency of human outdoor activities, and enhance the likelihood of skin exposure, thereby elevating the risk of virus transmission [19,20,40]. Conversely, decreased precipitation or drought conditions may reduce the water flow, increase the degree of eutrophication, and foster more suitable water pools for mosquito breeding and development [40,41]. This leads to an increase in mosquito populations and an elevated risk of human WNV transmission [42]. The harsh environmental conditions of high temperatures and drought can also force WNV host species to congregate in areas with more abundant or easily accessible food resources, such as urban areas and water sources. This can also increase the contact between hosts and WNV, thereby elevating the risk of virus transmission [43].

However, this study has some limitations. The number of WND cases obtained from the CDC might be an underestimate of the actual human cases. In particular, different states may employ different approaches when gathering case data. To partly control for the differences in surveillance methods among states, the state was included as a random effect in the analyses [44,45]. Some socioeconomic (e.g., human behaviors) or biotic factors (e.g., mosquito density and host community composition) may also be important in determining the spatial patterns of human WND risk. However, these factors were not included in the present analyses due to the lack of landscape-scale data for these factors. In addition, we did not consider the interaction effects of fixed variables and random factors due to the lack of specific related hypotheses and the large number of predictors (or levels of random factors). However, the conditional R^2^ of the best models for both regions was higher than 0.65, indicating that our models generally had good performance. Finally, as this study only focused on the correlations between the WND risk and the predictors, which may not disclose the causality underlying the detected relationships, future studies could explore the underlying mechanisms with additional field data, such as the bird community composition or the fluctuation of mosquito density. Despite these limitations, to the best of our knowledge, this study is among the first to investigate the effect of habitat fragmentation on the human risk of WND.

## 5. Conclusions

The present study investigated and compared the spatial patterns of the human risk of WND in the western and eastern United States and explored the effects of landscape and climatic factors, with a focus on habitat fragmentation. Our results showed that the human WND risk was positively correlated with the cover of developed areas in both regions, while was positively correlated with the cover of cropland and woody wetlands only in the western region. In contrast, the fragmentation of forests facilitated WND risk in both regions, while the fragmentation of developed areas was negatively associated with WND risk in the eastern region. The differences in the detected associations in the two regions may be caused by different vector species or by different processes (i.e., host-related or vector-related differences) between the regions. Under the background of ongoing land use change, this study provides new insights into the regional risk factors for WND in the United States and sheds light on the effects of habitat fragmentation on animal disease risk.

## Figures and Tables

**Table 1:** Description of landscape and climatic predictors used in the model. X relates to the land cover class.

Predictors	Description	Unit
*Landscape factors*		
PLAND X	The percentage of area covered by a land cover class X	/
FN X	The fragmentation index of a land cover class X	/
*Climatic factors*		
PreMean	Annual mean precipitation	mm
TemMean	Annual mean temperature	°C
BIO9	Mean temperature of the driest quarter	°C
BIO10	Mean temperature of the warmest quarter	°C
BIO11	Mean temperature of the coldest quarter	°C
BIO17	Precipitation of the driest quarter	mm
BIO18	Precipitation of the warmest quarter	mm
BIO19	Precipitation of the coldest quarter	mm

**Table 2:** The results (standardized regression coefficient, b, Z statistic and *p* values) for the single-variable regression analyses on the predictors correlated with human West Nile Disease in the eastern and western United States.

Predictors	Western			Eastern		
	b	Z	*p*-Value	b	Z	*p*-Value
*Landscape predictors* ^1^						
PLAND21	0.211	10.01	<0.001 ***	0.059	3.24	0.001 **
PLAND22	0.206	11.87	<0.001 ***	0.248	12.50	<0.001 ***
PLAND23	0.188	11.97	<0.001 ***	0.357	18.99	<0.001 ***
PLAND24	0.081	5.14	<0.001 ***	0.012	0.61	0.54
PLAND41	−0.164	−7.55	<0.001 ***	−0.323	−14.31	<0.001 ***
PLAND42	−0.403	−21.32	<0.001 ***	0.13	6.58	<0.001 ***
PLAND43	−0.257	−13.80	<0.001 ***	−0.129	−6.20	<0.001 ***
PLAND71	0.139	7.80	<0.001 ***	0.155	9.46	<0.001 ***
PLAND82	0.274	13.32	<0.001 ***	0.101	4.36	<0.001 ***
PLAND90	−0.121	−6.42	<0.001 ***	0.063	3.12	0.002 **
FN21	−0.073	−3.35	0.001 **	−0.292	−12.36	<0.001 ***
FN22	0.006	0.21	0.831	−0.048	−1.85	0.064
FN23	−0.123	−4.19	<0.001 ***	−0.23	−7.39	<0.001 ***
FN24	0.016	0.66	0.511	−0.041	−1.61	0.108
FN41	0.158	10.22	<0.001 ***	0.083	5.17	<0.001 ***
FN42	0.157	9.18	<0.001 ***	0.095	5.95	<0.001 ***
FN43	0.186	11.32	<0.001 ***	0.14	8.41	<0.001 ***
FN71	−0.188	−9.89	<0.001 ***	−0.06	−3.84	<0.001 ***
FN82	-0.078	−4.40	<0.001 ***	−0.005	−0.37	0.711
FN90	0.194	11.68	<0.001 ***	0.02	1.01	0.313
*Climatic predictors* ^2^						
TemMean	0.421	3.24	0.001 **	0.196	1.83	0.067
PreMean	−0.45	−20.89	<0.001 ***	−0.177	−5.33	<0.001 ***
BIO9	−0.094	−2.58	0.01 *	0.278	6.82	<0.001 ***
BIO10	0.574	19.78	<0.001 ***	0.491	11.30	<0.001 ***
BIO11	−0.137	−3.25	0.001 **	0.154	2.83	0.005 **
BIO17	−0.396	−16.93	<0.001 ***	−0.035	−0.89	0.373
BIO18	−0.447	−13.32	<0.001 ***	−0.222	−9.28	<0.001 ***
BIO19	−0.498	−20.56	<0.001 ***	0.195	4.00	<0.001 ***

Note: * *p* < 0.05; ** *p* < 0.01, *** *p* < 0.001; ^1^ PLAND_X, The percentage of area of a land cover class X, X—(21, developed open space; 22, developed low-intensity space; 23, developed medium-intensity space; 24, developed high-intensity space; 41, deciduous forest; 42, evergreen forest; 43, mixed forest; 71, grassland; 82, cropland; 90, woody wetlands); FNX, Fragmentation index for a land cover class X. ^2^ TemMean, the annual mean temperature; PreMean, the mean precipitation; Mean temperature of the driest quarter (BIO9), of the warmest quarter (BIO10), and of the coldest quarter (BIO11); Precipitation of the driest quarter (BIO17), of the warmest quarter (BIO18), and of the coldest quarter (BIO19).

**Table 3:** The results (standardized regression coefficient, b ± S.E., Z statistic and corresponding *p*-values) for the multi-variable regression analyses on the predictors correlated with human West Nile Disease in the eastern and western United States.

Predictors	Western			Eastern		
	b ± S.E.	Z	*p*-Value	b ± S.E.	Z	*p*-Value
AREA	0.29 ± 0.03	11.26	<0.001 ***	0.22 ± 0.02	9.62	<0.001 ***
Population	1.04 ± 0.04	27.73	<0.001 ***	0.75 ± 0.03	24.89	<0.001 ***
PLAND22	0.191 ± 0.021	8.95	<0.001 ***	/	/	/
PLAND23	/	/	/	0.24 ± 0.02	12.33	<0.001 ***
PLAND24	0.054 ± 0.018	3.06	0.002 **	/	/	/
PLAND41	0.055 ± 0.026	2.16	0.031 *	−0.19 ± 0.027	−6.96	<0.001 ***
PLAND42	−0.027 ± 0.028	−0.96	0.34	−0.075 ± 0.027	−3.29	0.001 **
PLAND43	−0.212 ± 0.025	−8.66	<0.001 ***	0.002 ± 0.027	0.06	0.95
PLAND71	/	/	/	0.146 ± 0.021	6.92	<0.001 ***
PLAND82	0.279 ± 0.028	9.86	<0.001 ***	/	/	/
PLAND90	0.266 ± 0.024	11.17	<0.001 ***	0.012 ± 0.023	0.53	0.59
FN21	0.006 ± 0.024	0.24	0.814	−0.23 ± 0.024	−9.44	<0.001 ***
FN23	−0.033 ± 0.031	−1.10	0.271	−0.18 ± 0.031	−5.86	<0.001 ***
FN41	0.01 ± 0.02	0.52	0.603	0.047 ± 0.02	2.33	0.020 *
FN42	−0.016 ± 0.02	−0.83	0.404	0.023 ± 0.018	1.29	0.20
FN43	0.048 ± 0.019	2.51	0.012 *	0.11 ± 0.021	5.10	<0.001 ***
FN71	/	/	/	−0.048 ± 0.016	−2.90	0.004 **
FN82	0.044 ± 0.019	2.36	0.018 *	/	/	/
FN90	0.125 ± 0.019	6.45	<0.001 ***	/	/	/
BIO10	0.317 ± 0.041	7.83	<0.001 ***	0.36 ± 0.05	7.50	<0.001 ***
BIO18	−0.365 ± 0.047	−7.83	<0.001 ***	−0.26 ± 0.03	−9.97	<0.001 ***
TemMean	0.318 ± 0.14	2.27	0.023 *	0.068 ± 0.09	0.77	0.41
PreMean	−0.18 ± 0.033	−5.38	<0.001 ***	/	/	/

Note: * *p* < 0.05; ** *p* < 0.01, *** *p* < 0.001; PLAND_X, The percentage of area of a land cover class X, X—(22, developed low-intensity space; 23, developed medium-intensity space; 24, developed high-intensity space; 41, deciduous forest; 42, evergreen forest; 43, mixed forest; 71, grassland; 82, cropland; 90, woody wetlands); FNX, Fragmentation index for a land cover class X. TemMean, the annual mean temperature; PreMean, the mean precipitation; BIO10, Mean temperature of the warmest quarter; BIO18, Precipitation of the warmest quarter.

## Data Availability

West Nile data was collected from CDC (https://www.cdc.gov/west-nile-virus/data-maps/historic-data.html) (accessed on 25 May 2023).

## Supplementary Materials

The following supporting information can be downloaded at: https://www.mdpi.com/article/10.3390/biology14030224/s1, Table S1. Summary of the number of human West Nile disease cases in the United States. Figure S1: Model validation results for the top model in eastern (first row) and western United States (second row). Figure S2: Partial plots for important predictors of the best multi-variable regression model for the western United States. Figure S3: Partial plots for important predictors of the best multi-variable regression model for the eastern United States.
