# Supplementary material for: Impacts of Urbanization and Habitat Characteristics on the Human Risk of West Nile Disease in the United States
Source: Biology (Basel). 2025 Feb 20;14(3):224. doi: 10.3390/biology14030224 (PMC11939350; doi:10.3390/biology14030224)

Table S1. Summary of the number of human West Nile disease cases in the United States.

Region	State	Number of cases
Eastern United States	Alabama	310
Eastern United States	Arkansas	299
Eastern United States	Connecticut	133
Eastern United States	Delaware	51
Eastern United States	District of Columbia	80
Eastern United States	Florida	353
Eastern United States	Georgia	432
Eastern United States	Illinois	1750
Eastern United States	Indiana	417
Eastern United States	Iowa	559
Eastern United States	Kentucky	109
Eastern United States	Louisiana	1492
Eastern United States	Maine	4
Eastern United States	Maryland	344
Eastern United States	Massachusetts	175
Eastern United States	Michigan	692
Eastern United States	Minnesota	771
Eastern United States	Mississippi	1236
Eastern United States	Missouri	452
Eastern United States	New Hampshire	7
Eastern United States	New Jersey	270
Eastern United States	New York	783
Eastern United States	North Carolina	79
Eastern United States	Ohio	604
Eastern United States	Pennsylvania	624
Eastern United States	Rhode Island	21
Eastern United States	South Carolina	104
Eastern United States	Tennessee	270
Eastern United States	Vermont	15
Eastern United States	Virginia	229
Eastern United States	West Virginia	20
Eastern United States	Wisconsin	284
Western United States	Arizona	1757
Western United States	California	6790
Western United States	Colorado	5499
Western United States	Idaho	1355
Western United States	Kansas	630
Western United States	Montana	606
Western United States	Nebraska	3803
Western United States	Nevada	351
Western United States	New Mexico	612
Western United States	North Dakota	1880
Western United States	Oklahoma	779
Western United States	Oregon	180
Western United States	South Dakota	2514
Western United States	Texas	5349
Western United States	Utah	424
Western United States	Washington	112
Western United States	Wyoming	755

Figure S1: Model validation results for the top model in eastern (first row) and western United States (second row)

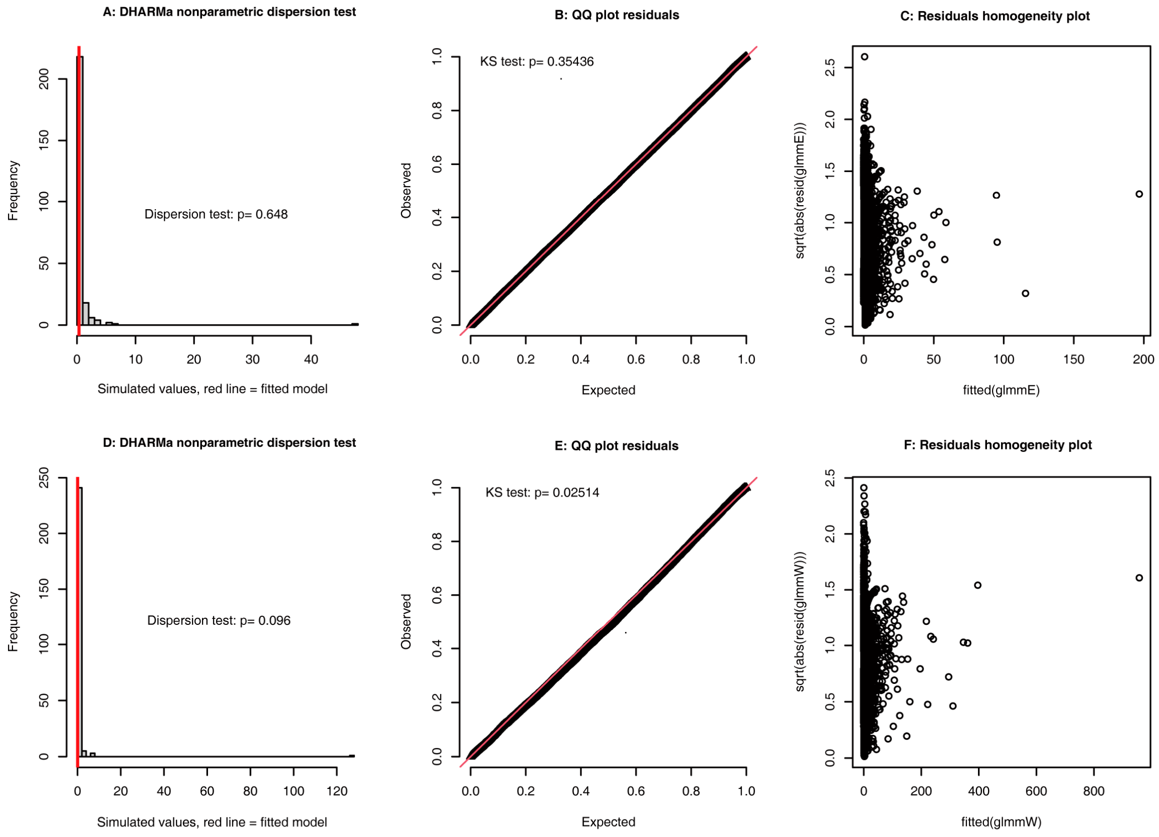

Figure S2: Partial plots for important predictors of the best multi-variable regression model for the western United States.

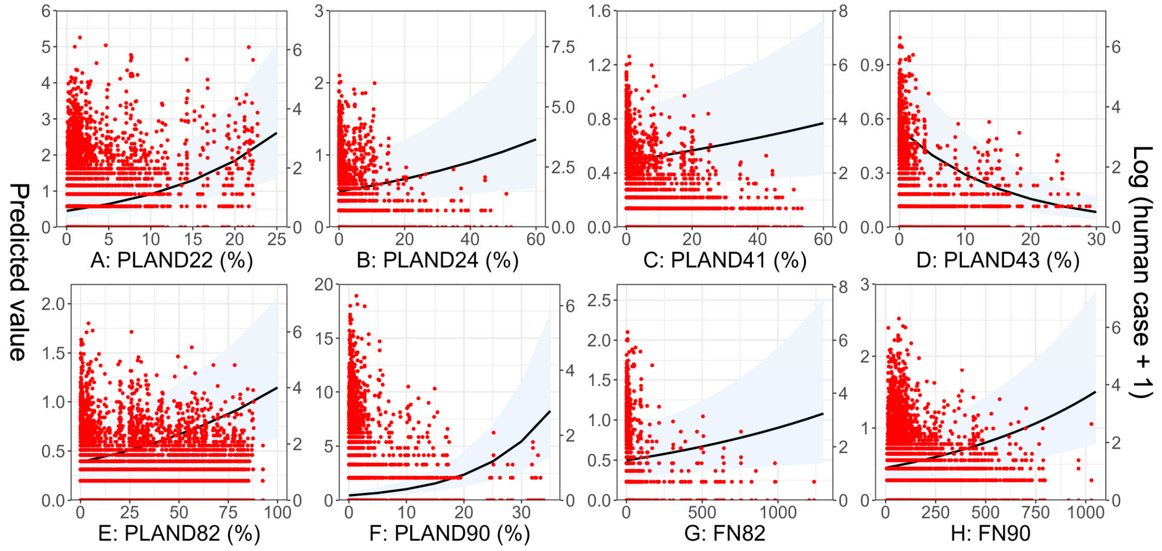

Figure S3: Partial plots for important predictors of the best multi-variable regression model for the eastern United States.

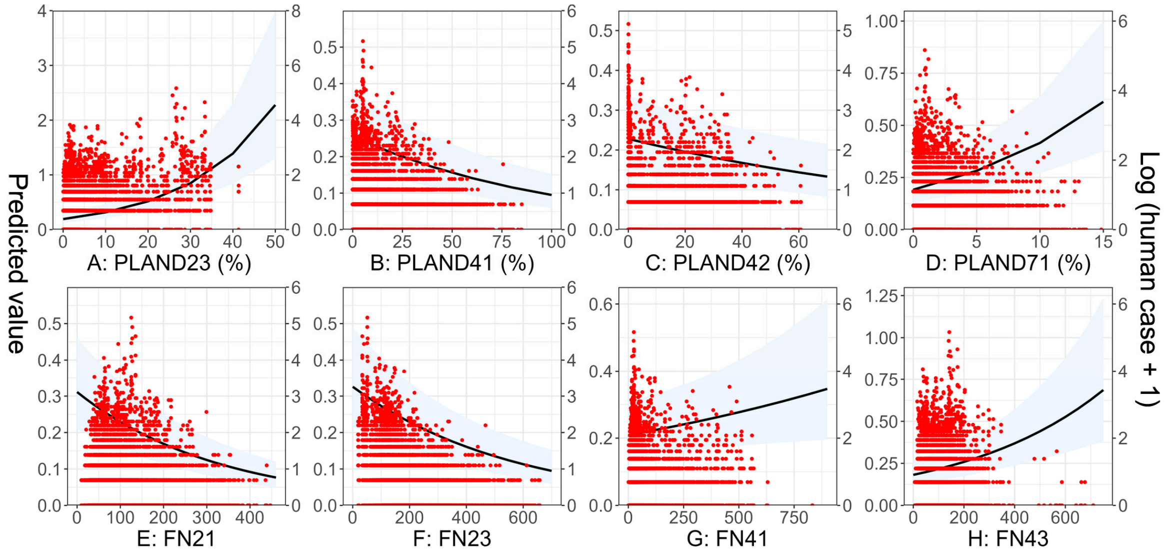

Supplement: Supplementary file 1 [file biology-14-00224-s001.zip › biology-3414263-supplementary.pdf]
